# Pias3 is necessary for dorso-ventral patterning and visual response of retinal cones but is not required for rod photoreceptor differentiation

**DOI:** 10.1242/bio.024679

**Published:** 2017-05-11

**Authors:** Christie K. Campla, Hannah Breit, Lijin Dong, Jessica D. Gumerson, Jerome E. Roger, Anand Swaroop

**Affiliations:** 1 Neurobiology-Neurodegeneration and Repair Laboratory, National Eye Institute, National Institutes of Health, 6 Center Drive, Bethesda, MD 20892, USA; 2 Nuffield Laboratory of Ophthalmology, Nuffield Department of Clinical Neurosciences, University of Oxford, Oxford OX3 9DU, UK; 3 Genetic Engineering Core, National Eye Institute, National Institutes of Health, Bethesda, MD 20892, USA; 4 Centre d'Etude et de Recherches Thérapeutiques en Ophtalmologie, Retina France, Orsay 91405, France; 5 Paris-Saclay Institute of Neuroscience, CNRS, Université Paris-Sud, Université Paris-Saclay, Orsay 91405, France

**Keywords:** Cell type specification, Gene regulation, Mouse knockout, Retina development, SUMOylation, Vision

## Abstract

Protein inhibitor of activated Stat 3 (Pias3) is implicated in guiding specification of rod and cone photoreceptors through post-translational modification of key retinal transcription factors. To investigate its role during retinal development, we deleted exon 2-5 of the mouse *Pias3* gene, which resulted in complete loss of the Pias3 protein. *Pias3^−/−^* mice did not show any overt phenotype, and retinal lamination appeared normal even at 18 months. We detected reduced photopic b-wave amplitude by electroretinography following green light stimulation of postnatal day (P)21 *Pias3^−/−^* retina, suggesting a compromised visual response of medium wavelength (M) cones. No change was evident in response of short wavelength (S) cones or rod photoreceptors until 7 months. Increased S-opsin expression in the M-cone dominant dorsal retina suggested altered distribution of cone photoreceptors. Transcriptome profiling of P21 and 18-month-old *Pias3^−/−^* retina revealed aberrant expression of a subset of photoreceptor genes. Our studies demonstrate functional redundancy in SUMOylation-associated transcriptional control mechanisms and identify a specific, though limited, role of Pias3 in modulating spatial patterning and optimal function of cone photoreceptor subtypes in the mouse retina.

## Abstract (precis)

**Summary:** Loss of Pias3 in mice results in altered dorso-ventral patterning of retinal cone photoreceptors by modulating the expression of a subset of genes, but does not affect rod development.

## INTRODUCTION

The vertebrate retina is designed to maximize the capture, integration, and transmission of visual information and consists of a stratified architecture with three cellular layers that include six neuronal cell types (Lamb, 2013). The response to light is initiated by rod and cone photoreceptors, which are distinguished by the type of opsin visual pigment they possess. Rod photoreceptors contain rhodopsin and can respond to even a single photon, thereby mediating dim light vision. In contrast, cone subtypes are defined by opsin pigments of distinct spectral properties and mediate daylight and color vision. Recent studies indicate that evolution of rod-dominance provided adaptive advantage to early mammals (Kim et al., 2016a). The spatial distribution of cone subtypes varies by species (Viets et al., 2016). Only about 3% of photoreceptors in the mouse retina are cones, with two subtypes that express opsins maximally sensitive to medium (M-opsin) or short (S-opsin) wavelengths of light (Nikonov et al., 2006). M- and S-opsin expression exhibits a dorsoventral gradient in the mouse retina, with most cones expressing varying amounts of both visual pigments (Applebury et al., 2000; Bumsted and Hendrickson, 1999).

The differentiation of photoreceptors from multipotent retinal progenitor cells is orchestrated by the combinatorial and synergistic or antagonistic action of a small number of transcription factors (Brzezinski and Reh, 2015; Cepko, 2014; Swaroop et al., 2010). Retinal progenitors expressing Otx2 have the potential to differentiate into bipolar or photoreceptor cells, whose fates are further restricted by Vsx2 and Prdm1, respectively (Brzezinski and Reh, 2015). The post-mitotic precursors expressing downstream factors, such as Crx, Rorβ, Nrl, and Nr2e3, differentiate as rod photoreceptors, whereas those expressing Crx and Rorβ develop as S-cones by default unless they are redirected to an M-cone fate by Trβ2 and Rxrγ (Ng et al., 2001, 2011; Roberts et al., 2005; Swaroop et al., 2010; Oh et al., 2008). Differential patterning of opsins is not induced until several days after the expression of these regulatory factors, suggesting that additional downstream mechanisms are needed to establish the dorsoventral expression gradient of M- and S-opsin pigments (Ng et al., 2001; Onishi et al., 2010; Roberts et al., 2005).

Post-translational modifications, such as phosphorylation and SUMOylation, can modulate the activity of transcription factors (Seet et al., 2006), including the two key photoreceptor-specific transcription factors Nr2e3 and Nrl (Roger et al., 2010; Swain et al., 2001). SUMOylation is a reversible modification involving the conjugation of SUMO protein to lysine residues and is associated with changes in the localization and/or function of target proteins (Chymkowitch et al., 2015; Geiss-Friedlander and Melchior, 2007; Lyst and Stancheva, 2007). Several SUMO pathway genes are expressed in the retina and implicated in photoreceptor development and disease (Abad-Morales et al., 2015). E3 SUMO ligase protein inhibitor of activated STAT 3 (Pias3) is reported to regulate both rod and cone subtype differentiation (Onishi et al., 2009, 2010). Pias3 can augment Trβ-dependent activation of M-opsin promoter, with concurrent repression of Rorα-mediated S-opsin promoter (Onishi et al., 2010). Pias3 can also SUMOylate Nr2e3 to maximally repress cone-specific gene transcription (Onishi et al., 2009). Together, these results suggested a dual role of Pias3 in rod and cone photoreceptor development through modulation of distinct targets in each cell type.

We generated a null allele in mice by targeting the *Pias3* gene (*Pias3^−/−^*) to elucidate Pias3 function during development. Despite a demonstrated role of Pias3 in multiple cellular pathways (Sundvall et al., 2012; Wu and Zou, 2016; Yagil et al., 2010), *Pias3^−/−^* mice exhibited no gross abnormality and lack of Pias3 did not have a dramatic impact on retinal development and photoreceptor differentiation. Nonetheless, *Pias3^−/−^* mice exhibited altered dorsoventral gradient of S-opsin, reduced M-cone-mediated visual response, and misregulation of a subset of vision-related genes, highlighting a specific role of Pias3 in establishing dorsoventral patterning and visual response of cone photoreceptors in the mouse retina.

## RESULTS AND DISCUSSION

**Fig. 1.**
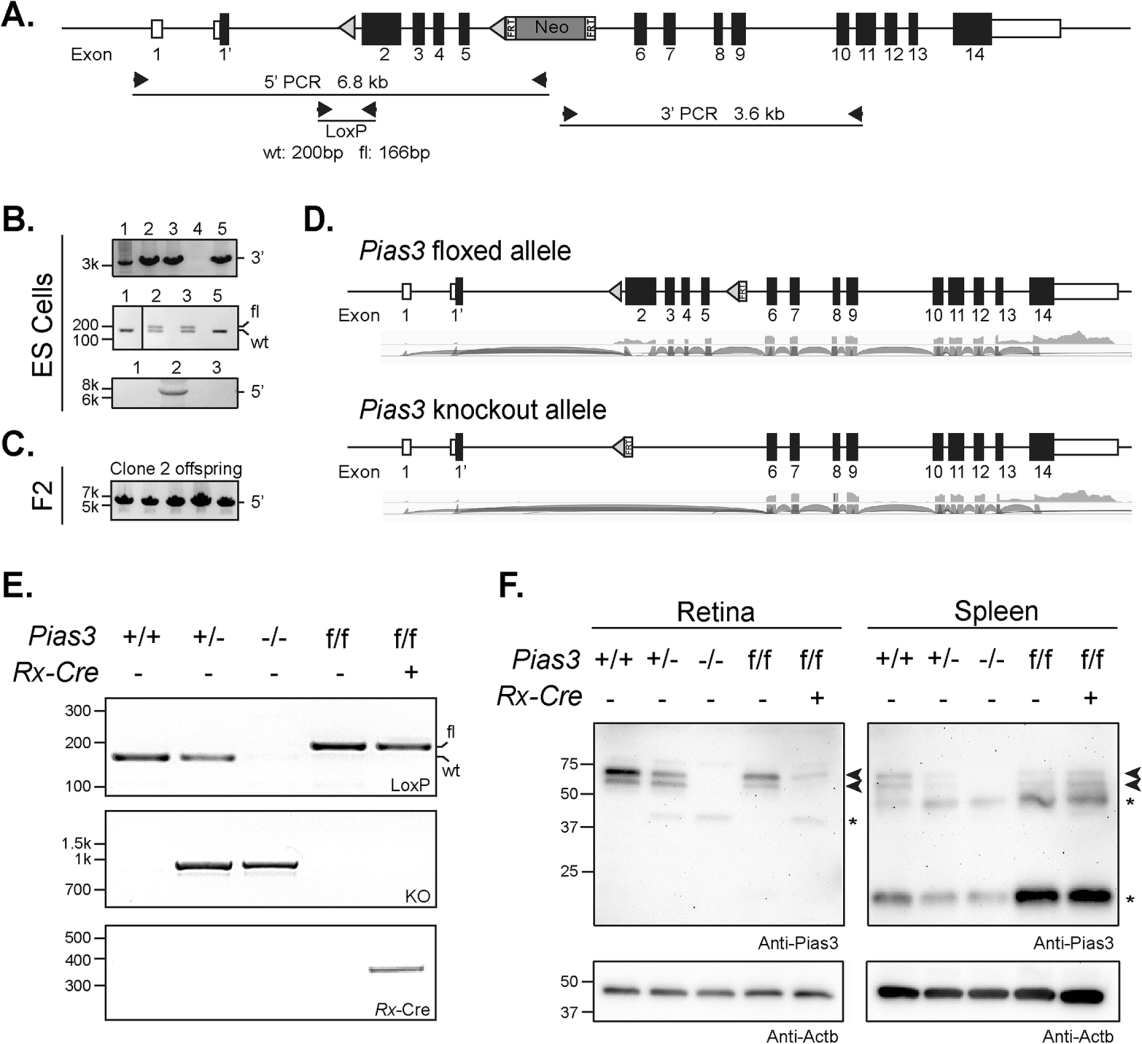
**Targeted disruption of mouse *Pias3*.** (A) Strategy for targeting *Pias3*. Targeting vector includes LoxP sites (triangles) flanking exons 2 to 5 of the *Pias3* gene and neomycin cassette (Neo) enclosed by FRT sites. Arrows indicate position of PCR primers. (B) PCR screening of correctly targeted ES cells. Presence of the neomycin cassette in ES cell lines was confirmed by a 3.6 kb product (3′-PCR). Floxed (fl) or wild-type (wt) 5′ LoxP sites were distinguished by a 200 or 166 bp product, respectively. Presence of LoxP sites and neomycin cassette was further confirmed by 6.8 kb product using 5′-PCR. (C) 5′ PCR genotyping of F2 generation mice. Presence of LoxP sites and neomycin cassette in the genome of offspring derived from ES clone 2 was validated by a 6.8 kb product (5′-PCR). (D) *Pias3* mRNA expression in mouse retina. RNA-Seq was performed using P21 *Pias3^+/+^* and *Pias3^−/−^* retina. Sashimi plots of raw read alignments are shown corresponding to *Pias3* floxed and knockout alleles. (E) PCR analysis of genomic DNA. Presence of wild-type (wt), floxed (fl), knockout (KO), and *Rx-*Cre alleles in the genome were confirmed by PCR. (F) Immunoblot analysis of protein extracts from conditional and complete knockout mice. Immunoblots of retina and spleen protein extracts from each mouse line were probed with anti-Pias3 antibody and anti-Actb as a loading control. Arrowheads represent endogenous Pias3 protein isoforms. Asterisks indicate nonspecific staining (spleen) and/or aberrant protein isoforms arising from the deletion of exons 2-5 (retina). PCR and immunoblot results are representative of at least three experimental replicates.

**Fig. 2.**
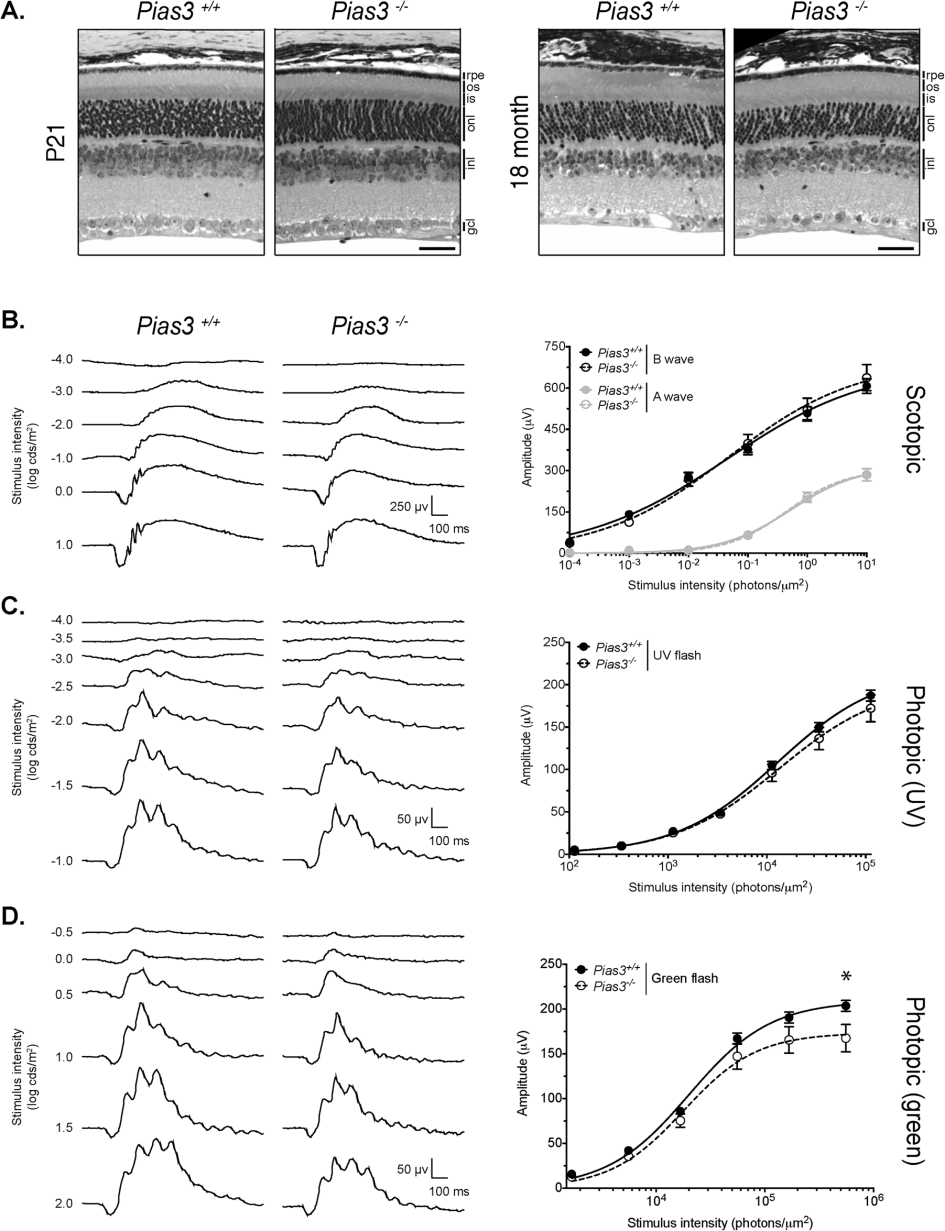
**Normal retinal morphology but reduced ERG responses in *Pias3^−/−^* mice.** (A) Hematoxylin and eosin (H&E) staining of methacrylate sections. Overall histology was assessed by H&E staining of retina sections from P21 and 18-month-old mice (*n*≥2 of each age and genotype). Scale bar: 50 μm. (B) Representative scotopic ERGs for *Pias^+/+^* and *Pias3^−/−^* mice. P21 mice were dark-adapted for 24 h and scotopic responses recorded. Intensity response curves of the average a- and b-wave responses of ten *Pias^+/+^* and six *Pias3^−/−^* mice (mean±s.e.m.) are shown. (C) Representative S-cone ERGs for *Pias^+/+^* and *Pias3^−/−^.* P21 mice were light adapted and responses to UV light flashes were recorded. Intensity response curves of the average b-wave responses of ten *Pias^+/+^* and six *Pias3^−/−^* mice (mean±s.e.m.) are shown. (D) Representative M-cone ERGs for *Pias^+/+^* and *Pias3^−/−^* mice. P21 mice were light-adapted and responses to green light flashes were recorded. Intensity response curves of the average b-wave responses of ten *Pias^+/+^* and six *Pias3^−/−^* mice (mean±s.e.m.; *P=0.0158) are shown.

**Fig. 3.**
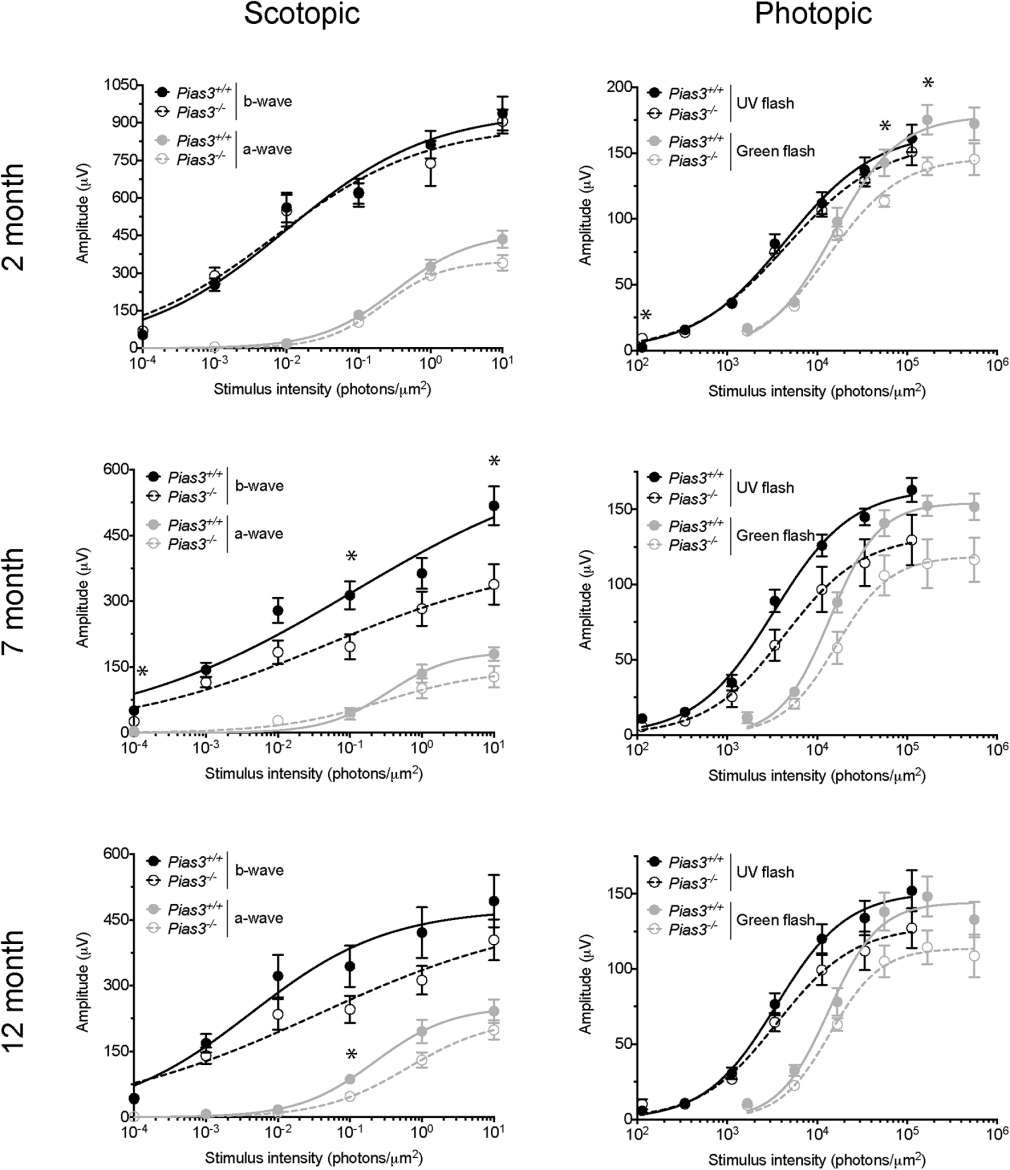
***Pias3^−/−^* mice exhibit age-associated decline in dark- and light-adapted flash ERG responses.** Mice at 2, 7, and 12 months of age were dark-adapted for 24 h before recording scotopic responses. Intensity response curves of the average a- and b-wave responses of three mice of each genotype (mean±s.e.m.) are shown in the left panel. Mice were then light-adapted and responses to UV and green light flashes were recorded. Intensity response curves of the average photopic b-wave response (mean±s.e.m.) are shown in the right panel. *P<0.05.

A targeting vector with LoxP sites spanning exon 2 to 5 of the *Pias3* gene and neomycin selection marker flanked by FRT sites was used to establish a germline knockout mouse line on C57BL/6J background (*Pias3^−/−^*) (see Fig. 1 and Materials and Methods for details). *Pias3^−/−^* mice were viable and fertile despite complete loss of Pias3 protein in all tissues examined. Histological analysis of the *Pias3^−/−^* retina using hematoxylin and eosin (H&E) staining of methacrylate sections revealed proper lamination and thickness of retinal cell layers, including photoreceptors, in both young [postnatal day (P)21] and old (18 month) mice (Fig. 2A). Given that *Pias3* was reported to control photoreceptor development (Onishi et al., 2009, 2010), we assessed visual function in *Pias3^−/−^* mice by electroretinography (ERG). Scotopic (rod-mediated) and UV (S-cone mediated) responses of *Pias3^−/−^* retina did not differ significantly from the wild-type at P21 and begin to decline only by 7 months (Fig. 2B,C and Fig. 3). However, the maximum response of P21 *Pias3^−/−^* mice to green stimuli (M-cone mediated) was impaired [203.6±6.1 (mean±s.e.m.) versus 167.4±15.3 μv, *P*=0.0158] and remained so at least until 12 months (Figs 2D and 3). These results suggest an early and predominantly M-cone defect, with gradual decline of rod and S-cone function at older ages, in the absence of *Pias3*.

**Fig. 4.**
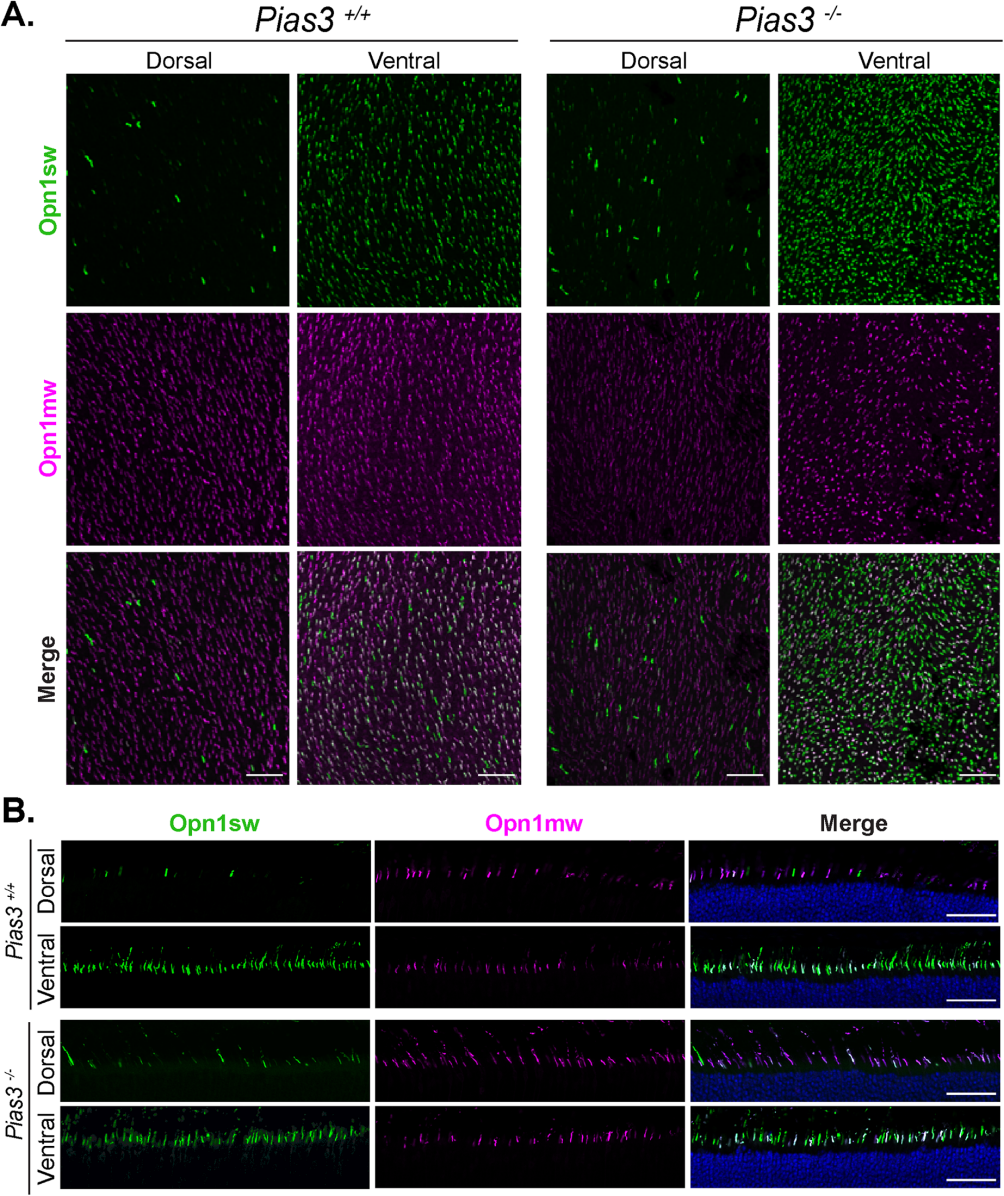
**Altered dorsoventral opsin expression in the *Pias3^−/−^* retina.** (A) Flat-mount staining for cone opsins in dorsal and ventral retina. Cone opsin expression in flat-mounted retina from P21 mice was detected by immunostaining against S-opsin (Opn1sw, green) and M-opsin (Opn1mw, magenta). Scale bar: 50 μm. (B) Section staining for cone opsins in dorsal and ventral retina. Cone opsin expression in frozen sectioned eyes from P21 mice was detected by immunostaining against S-opsin (Opn1sw, green) and M-opsin (Opn1mw, magenta). Nuclei were detected by DAPI (blue). Scale bar: 50 μm. Results are representative of at least three biological replicates.

**Fig. 5.**
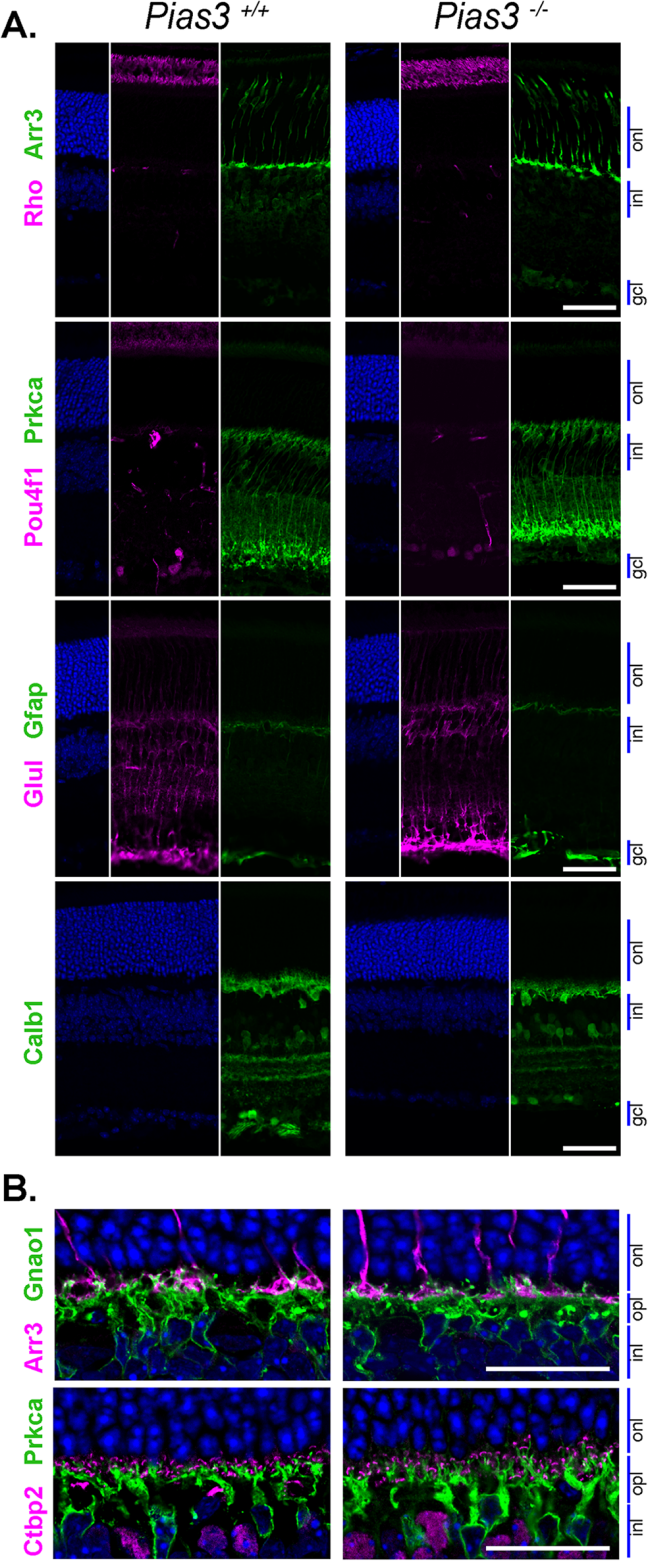
**Retinal lamination and individual cell morphologies in *Pias3^−/−^* mice.** (A) Immunostaining of P21 mouse retina sections. Primary antibodies were used to detect rod outer segments (Rho), cones (Arr3), ganglion cells (Pou4f1), bipolar cells (Prkca), normal and activated Müller glia (Glul and Gfap, respectively), and horizontal and amacrine cells (Calb1). Scale bar: 50 μm. (B) Immunostaining of outer plexiform layer synapses. Morphologies of cone synapse (Arr3), ON-bipolar synapse (Gnao1), ribbon synapse (Ctbp2), and rod bipolar synapse (Prkca) were examined by immunostaining using frozen retina sections from P21 mice. Scale bar: 25 μm. Results are representative of at least three biological replicates.

Immunohistochemical (IHC) staining of P21 *Pias3^−/−^* retina showed altered dorsoventral gradient of S-opsin, with more S-opsin-positive cones identified in the dorsal region of both flat-mounted (Fig. 4A) and sectioned (Fig. 4B) retina. To determine whether these opsins were expressed in cones rather than ectopically in rod photoreceptors, further staining was performed with peanut agglutinin (PNA) to specifically examine cone outer matrix sheaths. All S- and M-opsin staining was consistently localized within cone outer segments in *Pias3^−/−^* retina (data not shown). A series of IHC stainings did not show any significant difference between wild-type (*Pias3^+/+^*) and *Pias3^−/−^* retina for markers of rods (Rhodopsin, Rho), cones (Cone arrestin, Arr3), Müller glia (Glutamine synthetase, Glul), activated Müller glia (Glial fibrillary acidic protein, Gfap), bipolar cells (Protein kinase C alpha, Prkca), ganglion cells (POU class 4 homeobox 1, Pou4f1 or Brn3a), or amacrine and horizontal cells (Calbindin 1, Calb1) (Fig. 5A). Since reduced ERG b-wave responses could result from a synaptic transmission defect, we performed immunostaining with synaptic markers – Ctbp2 (C-terminal binding protein 2, ribeye)+Prkca and Arr3+Gnao1 (G protein subunit alpha O1) (Fig. 5B). No apparent abnormality in photoreceptor-bipolar synapses suggested that reduced photoreceptor response in *Pias3^−/−^* retina is caused by subtle changes within neurons and not by defects in cell fate specification or morphogenesis. A similar series of IHC was performed in 18-month-old *Pias3^−/−^* retina and did not reveal any differences with advanced age (Fig. S1).

**Fig. 6.**
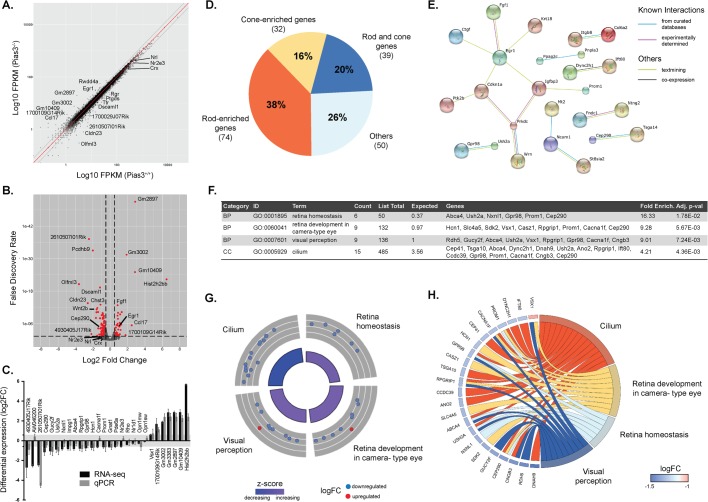
**RNA-seq analysis of P21 *Pias3^−/−^* retina.** (A) Scatter plot of global gene expression profiles between *Pias3^+/+^* and *Pias3^−/−^* retina. RNA expression (in FPKM) of each gene expressed in *Pias3^+/+^* (*x*-axis) is plotted against those in *Pias3^−/−^* (*y*-axis) retina (log_10_ scale). Red line represents equal expression value between samples. Gray lines represent FC of 1.5. (B) Volcano plot of differentially expressed genes in *Pias3^−/−^* retina. Difference in RNA expression between *Pias3^−/−^* and *Pias3^+/+^* retina genes is plotted on the *x*-axis (log_2_ scale), and FDR adjusted significance is plotted on the *y*-axis. Genes up- or down-regulated by a factor ≥1.5 with FDR ≤0.05 are indicated in red. Vertical dashed lines represent FC=1.5. (C) Validation of RNA-seq results by qPCR. Differential expression values were compared between RNA-seq (black) and qPCR (dark gray) for 28 genes of either undefined or eye-related functions. Error bars represent s.e.m.; light gray background represents a FC of 1.5. (D) Classification of differentially expressed genes (DEGs) by cell type. DEGs were identified as rod- and/or cone-enriched by meta-analysis using RNA-Seq data from flow-sorted rods and cone-like photoreceptors. (E) Interaction analysis of DEGs. STRING (Search Tool for the Retrieval of Interacting Genes/Proteins) analysis was used to map putative protein interactions between DEGs. (F) Gene ontology (GO) annotations of DEGs. The top four over-represented GO pathways amongst DEGs at P21 were identified by GO enrichment analysis generated by PANTHER. (G) Circular visualization of GO enrichment analysis. Down-regulated genes (blue dots) and up-regulated genes (red dots) within each GO pathway are plotted based on logFC. Z-score bars indicate if an entire biological process is more likely to be increased or decreased based on the genes within it. (H) Chord plot representation of DEGs related to GO annotations. Overlaps in GO annotation amongst genes within each category are visualized.

**Fig. 7.**
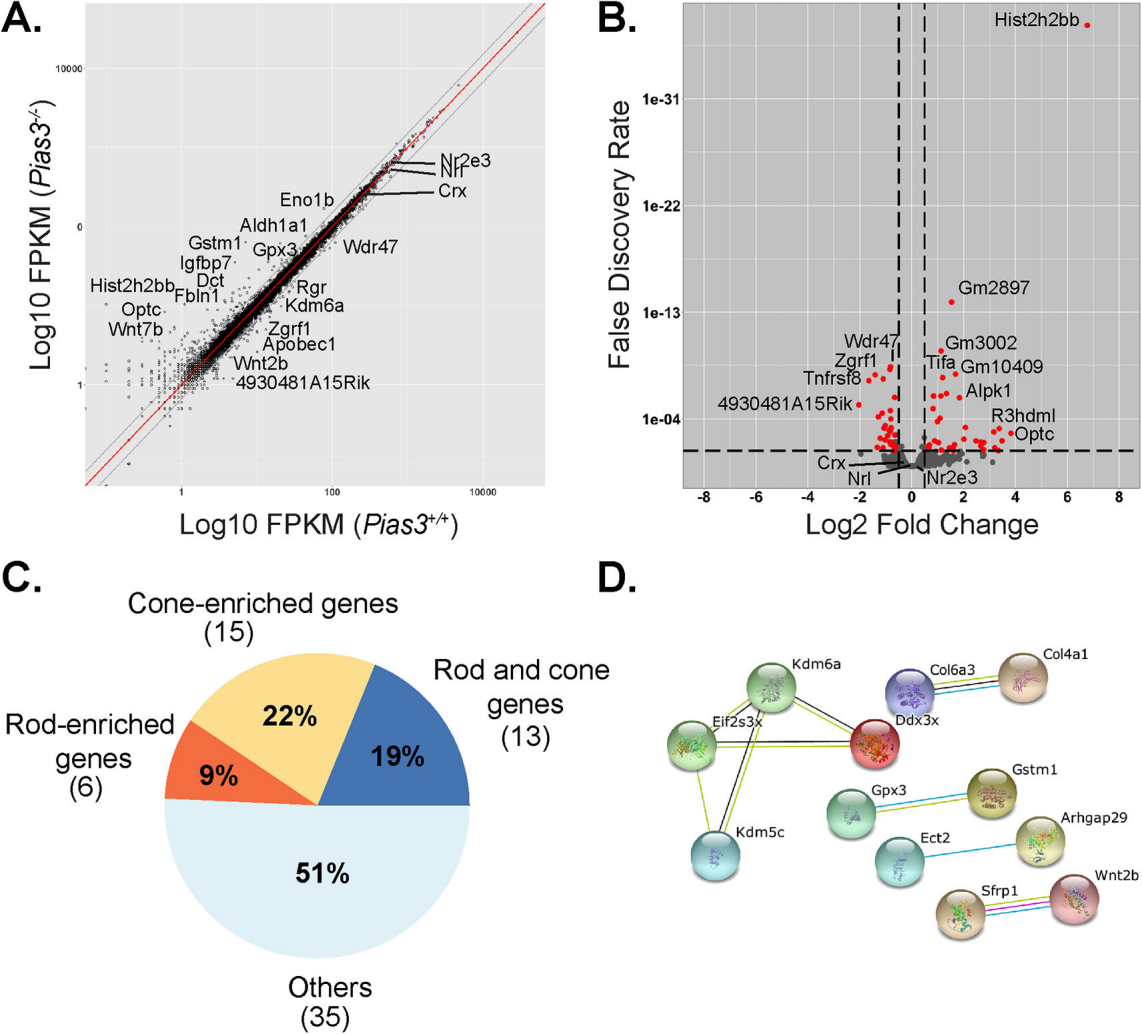
**RNA-Seq analysis of 18-month-old *Pias3^−/−^* mouse retina.** (A) Scatter plot of global gene expression profiles of 18-month-old *Pias3^+/+^* and *Pias3^−/−^* retina. RNA expression (shown in FPKM) of all expressed genes in *Pias3^+/+^* (*x*-axis) retina was plotted against those in *Pias3^−/−^* (*y*-axis) retina (log_10_ scale). Red line represents equal expression value between samples. Gray lines represent FC of 1.5. (B) Volcano plot of differentially expressed genes in *Pias3^−/−^* retina. Fold change difference in RNA expression between *Pias3^−/−^* and *Pias3^+/+^* retina is plotted on the *x*-axis (log_2_ scale), and false discovery rate adjusted significance is shown on the *y*-axis. Genes up- or downregulated by a factor ≥1.5 with false discovery rate ≤0.05 are indicated in red. (C) Classification of differentially expressed genes (DEGs) by cell type. DEGs were identified as rod- and/or cone-enriched by meta-analysis using RNA-Seq data from flow-sorted rods and cone-like photoreceptors. (D) Interaction analysis of DEGs. STRING (Search Tool for the Retrieval of Interacting Genes/Proteins) analysis was used to map putative protein interactions between DEGs.

Next, we performed whole transcriptome analysis (RNA-Seq) of young (P21) and aged (18 month) wild-type and *Pias3^−/−^* retina (Figs 6 and 7). Using a fold change (FC) cutoff of 1.5, FPKM (fragments per kilobase of exon per million reads mapped) of ≥2, and a false discovery rate (FDR)≤0.05, we identified 195 differentially expressed genes (DEGs) at P21 (Fig. 6B; Table S1). We selected 28 uncharacterized or retina-related DEGs at P21 with FPKM >30 for qPCR validation and 24 of these were confirmed as being up- or down-regulated in both RNA-Seq and qPCR analyses (Fig. 6C). *Opn1mw* (M-opsin) and *Opn1sw* (S-opsin) were not identified as DEGs by our criteria though small differences were detected by qPCR (Fig. 6C). Using *Nrl^−/−^* and flow-sorted rod photoreceptor RNA-Seq data (Kim et al., 2016b), we observed several rod- and cone-enriched genes (38% and 16%, respectively) among the P21 *Pias3^−/−^* DEGs (Fig. 6D). Further analysis using STRING highlighted *Egr1* as a central node interacting with nine other genes (Fig. 6E). Pathway analysis of the 195 DEGs revealed an enrichment of genes related to retinal homeostasis, retina development, visual perception, and cilium (Fig. 6F). Circular visualization of genes in these biological processes indicated that most were downregulated in *Pias3^−/−^* retina (Fig. 6G) and some belonged to multiple pathways as shown by Chord plot representation (Fig. 6H). To determine whether changes in gene expression resulted from alterations in SUMOylation status of upstream transcription factors, we assessed global SUMOylation status of proteins in both *Pias3^+/+^* and *Pias3^−/−^* retina. No major difference was evident between SUMOylated proteins in the *Pias3^−/−^* retina by immunoblot analysis (Fig. S2). Using the same filtering criteria, whole transcriptome analysis of 18 month old *Pias3^−/−^* retina identified only 69 DEGs (Fig. 7B;Table S2), including fewer rod- and slightly more cone-enriched genes compared to P21 analysis (9% and 22%, respectively) (Fig. 7C). Furthermore, STRING analysis highlighted Kdm6a and Kdm5c, two histone demethylases, in relation with transcription/translation factors Ddx3x and Eif2s3x (Fig. 7D). The number of DEGs was too small to perform pathway analysis. Overall, the low number of identified DEGs in aging retina compared to P21 seems to reflect redundant mechanisms that may compensate the absence of *Pias3*.

Pias proteins function as E3 SUMO ligases that facilitate, but are not required for, the covalent linkage of SUMO groups to lysine residues of target proteins (Schmidt and Muller, 2003). SUMOylation plays a critical role in modifying target protein function by altering subcellular localization, protein-protein interaction, and transcriptional activity (Geiss-Friedlander and Melchior, 2007); for example, SUMOylation state can affect transcriptional activation of several retinal proteins such as Rorα, Trβ1, Trβ2, Nrl, and Nr2e3 (Hwang et al., 2009; Liu et al., 2012; Onishi et al., 2009, 2010; Roger et al., 2010) as well as protein interactions and stability of Gtf2ird1 and Phosducin (Klenk et al., 2006; Widagdo et al., 2012).

To our surprise, the consequence of Pias3 deletion was less pronounced in the knockout mouse retina compared to previous reports where *in vivo* electroporation was used to knockdown *Pias3* in developing photoreceptors (Onishi et al., 2009, 2010). We suggest that a mild phenotype in *Pias3^−/−^* retina is likely due to redundancy in the SUMOylation machinery and functional overlap with other Pias proteins, which have the potential to catalyze the addition of SUMO groups to the same targets (Rytinki et al., 2009). In *Pias3^−/−^* retina, the expression of *Pias* genes and other E3 SUMO ligases was unchanged, indicating that compensation is not due to increased expression at the transcriptional level but rather through functional redundancy (Table S3). In addition, analysis of published RNA-Seq data from flow-sorted photoreceptors of wild-type and cone-only retina showed that multiple E3 SUMO ligases are expressed in both rod and cone photoreceptors (Table S3) (Kim et al., 2016b). Such functional redundancy has been described previously. Indeed, Rorα can be SUMOylated by Pias3 as well as Pias2 and Pias4 (Hwang et al., 2009), and Trβ can also be SUMOylated by Pias1 (Liu et al., 2012). Compensatory mechanisms are especially feasible in a germline knockout mouse model where cells can overcome imbalances during development. Relatively mild phenotypes have also been observed for *Pias1^−/−^* (Liu et al., 2004), *Pias2^−/−^* (Santti et al., 2005), and *Pias4^−/−^* (Roth et al., 2004; Wong et al., 2004) mice, whereas embryonic lethality is observed in double *Pias1^−/−^/Pias4^−/−^* (Tahk et al., 2007) or SUMO-conjugating enzyme *Ubc9^−/−^* mice (Nacerddine et al., 2005). Breeding *Pias3^−/−^* mice with other available *Pias^−/−^* lines would likely result in more dramatic phenotypes by preventing compensatory mechanisms; however, tissue-specific or conditional deletions might be necessary to prevent early lethality.

The observation of an early M-cone defect in *Pias3^−/−^* retina is intriguing. Pias3 is expressed in M-cones (Onishi et al., 2010) and might serve as the primary E3 SUMO ligase in these cells to fine-tune the expression of a subset of genes. It would thus appear that rods and S-cone photoreceptors are better equipped to maintain appropriate gene expression pattern even in the absence of Pias3 by using alternative SUMO-conjugating enzymes (Table S3). This explanation could also account for higher S-opsin expression in the dorsal *Pias3^−/−^* retina, resulting from incomplete suppression of S-opsin in cells that are fated to be M-cones. Gene expression changes in M-cone population are likely masked because of their small numbers in the whole retina, and transcriptome profiling of isolated M- and S-cones from the *Pias3^−/−^* retina would be necessary to test this hypothesis.

The mild phenotype and seemingly unperturbed global SUMOylation status of *Pias3^−/−^* retina suggest that Pias3 reinforces or augments gene expression changes rather than controls broader cell fate or developmental decisions. We propose that the addition of SUMO groups to target proteins, such as Nrl and Nr2e3, by Pias3 is a transient and dynamic process that serves to modulate (enhance or suppress) their activity in a context-dependent manner and that additional SUMO-conjugating enzymes are able to compensate its function in *Pias3^−/−^* retina. Fine adjustments in gene expression patterns and redundancy in control mechanisms are necessary to establish and optimize spatial and functional organization of photoreceptors in the mammalian retina.

## MATERIALS AND METHODS

### Generation of *Pias3^−/−^* mice

A gene-targeting vector was constructed to add LoxP sites flanking exons 2 to 5 of the *Pias3* gene followed by *neo* cassette enclosed by FRT sites. Correctly targeted embryonic stem cell lines were identified by PCR at both ends designed to detect homologous recombination and used to generate chimeric mice. Original founders were mated twice with *Actin*-Flp recombinase to remove the *neo* cassette and were backcrossed to C57BL/6J mice. Specific deletion in the retina and the forebrain was achieved by crossing *Pias3^f/f^* mice with *Rx-*Cre-positive males (Swindell et al., 2006). However, leaky *Cre* expression resulted in recombination within the oocyte in a few lines tested, leading to complete deletion of *Pias3* exon 2-5 in all tissues and permitting us to establish a germline knockout mouse line (*Pias3^−/−^*). Wild-type and knockout mice of either sex were used for all experiments. All experiments were conducted according to protocols approved by a local Institutional Animal Care and Use Committee and adhered to the Association for Research in Vision and Ophthalmology Statement for animal use in ophthalmic and vision research.

### PCR genotyping

Genomic DNA was extracted from tail clippings and both wild-type and floxed alleles were detected via amplification using primers flanking either side of the 5′ LoxP site yielding products of 166 bp and 200 bp, respectively (5′ LoxP Insertion, Table S5). Alleles with *Pias3* exon 2-5 deletion were detected in the same manner but using a reverse primer within exon 6 of the gene to amplify a 1 kb product generated by the knockout allele (*Pias3*-KO Allele, Table S5). *Rx-Cre* was detected by amplification of a 350 bp fragment (*Rx-Cre*, Table S5).

### Immunoblotting

Retina and spleen tissues were isolated from mice at P21 and lysed by sonication in ice-cold radioimmunoprecipitation buffer supplemented with protease inhibitors (Roche Applied Science) and 20 mM N-ethylmaleimide (NEM) (Sigma-Aldrich). Supernatants were quantified by BCA protein assay according to manufacturer's protocol (Pierce) and solubilized in 4× Laemmli buffer+β-mercaptoethanol. After denaturation, 20 µg of the total protein extract was separated by SDS-PAGE and transferred to PVDF membrane (Bio-Rad). Standard immunoblot procedure was followed, as previously described (Roger et al., 2010).

### Immunohistochemistry

Eyeballs were enucleated and fixed in 4% PFA for 30 min prior to dissection. For cryosections, the eyecup was fixed for an additional 30 min and cryoprotected in 30% sucrose before being frozen in OCT embedding medium (Sakura Finetek USA, Torrance CA, USA) then cut 12 µm thick. For whole mounts, the retina was fixed for an additional 30 min. Both cryosections and whole mounts were blocked for 1 h at room temperature in blocking solution (5% normal donkey serum, 0.3% Triton X-100 in PBS), then incubated overnight at 4°C with primary antibodies (Table S4) diluted in blocking solution. The next day, they were washed 4× with 0.2% Triton X-100 in PBS then incubated at room temperature for 1 h with appropriate secondary antibodies (Table S4) diluted in blocking solution. After four subsequent washings, sections were stained for 5 min with DAPI in 1× PBS and washed again, mounted with Fluoromount-G (Southern Biotech, Birmingham AL, USA) and imaged using a Zeiss LSM 700 confocal microscope.

### Histology

Eyeballs were enucleated and fixed in 4% glutaraldehyde in PBS for 30 min, then transferred to 4% PFA in PBS until being embedded in glycol methacrylate. Five-micron-thick sections were cut, stained with H&E, and imaged using a Zeiss Axio Imager Z1 brightfield microscope.

### Quantitative PCR

Total RNA was extracted by Trizol LS reagent (Thermo Fisher), according to the manufacturer's protocol, and its quality was assessed using an Agilent 2100 bioanalyzer and RNA 6000 Nano chip. Only RNA samples with RNA integrity number (RIN) of ≥7.9 were subjected to first-strand cDNA synthesis using SuperScript II Reverse Transcriptase (Life Technologies). cDNA samples then served as templates for quantitative PCR, per Fast SYBR Green Master Mix manufacturer's protocol (Applied Biosystems) performed in biological and technical triplicates. Primer sequences are provided in Table S5 (*Hprt* used as internal control).

### RNA-seq analysis

Whole transcriptome analysis was performed using three independent biological replicates from retina of *Pias3^+/+^* and *Pias3^−/−^* mice at P21 and at 18 months of age. Total RNA was extracted by Trizol LS reagent (Thermo Fisher), according to the manufacturer's protocol, and its quality was assessed using an Agilent 2100 bioanalyzer and RNA 6000 Nano chip. Only RNA samples with RIN≥7.9 were used. Stranded RNA-seq libraries were constructed from 100 ng of total RNA using a modified TruSeq RNA Sample preparation kit protocol (Roger et al., 2014). Paired-end sequencing of 100 bases length was performed on HiSeq 2500 system (Illumina). Pass-filtered reads were mapped using TopHat v2.1.1 (Trapnell et al., 2009) and aligned to UCSC mouse reference genome mm10. Count table of the gene features was obtained using FeatureCounts (Liao et al., 2014). Normalization, differential expression analysis and FPKM values were computed using EdgeR (Robinson et al., 2010). An FPKM filtering cutoff of 2 in at least one of the 6 samples was applied. FDR≤0.05 was considered significant and a cutoff of fold change of 1.5 was applied to identify differentially expressed isoforms. R packages and JMP Software (SAS) were used for data mining. GO annotation and pathways enrichment analysis was performed using Panther Classification System (http://pantherdb.org/).

### Electroretinography

ERG responses to light stimulation were recorded simultaneously from both eyes on an Espion Electrophysiology System (Diagnosys LLC, Lowell, MA, USA) as previously described for both photopic and scotopic conditions (Ng et al., 2011). A minimum of six responses from each age/genotype to each light stimulus were recorded (80% power to detect an effect size of 53 μv assuming 30 μv standard deviation). Statistical analysis (two-tailed unpaired Student's *t*-test) was performed using GraphPad Prism 7 software.

## References

[BIO024679C1] Abad-Morales V., Domenech E. B., Garanto A. and Marfany G. (2015). mRNA expression analysis of the SUMO pathway genes in the adult mouse retina. *Biol. Open* 4, 224-232. 10.1242/bio.201410645 25617419 PMC4365491

[BIO024679C2] Applebury M. L., Antoch M. P., Baxter L. C., Chun L. L. Y., Falk J. D., Farhangfar F., Kage K., Krzystolik M. G., Lyass L. A. and Robbins J. T. (2000). The murine cone photoreceptor: a single cone type expresses both S and M opsins with retinal spatial patterning. *Neuron* 27, 513-523. 10.1016/S0896-6273(00)00062-3 11055434

[BIO024679C3] Brzezinski J. A. and Reh T. A. (2015). Photoreceptor cell fate specification in vertebrates. *Development* 142, 3263-3273. 10.1242/dev.127043 26443631 PMC4631758

[BIO024679C4] Bumsted K. and Hendrickson A. (1999). Distribution and development of short-wavelength cones differ between Macaca monkey and human fovea. *J. Comp. Neurol.* 403, 502-516. 10.1002/(SICI)1096-9861(19990125)403:4<502::AID-CNE6%3.0.CO;2-N 9888315

[BIO024679C5] Cepko C. (2014). Intrinsically different retinal progenitor cells produce specific types of progeny. *Nat. Rev. Neurosci.* 15, 615-627. 10.1038/nrn3767 25096185

[BIO024679C6] Chymkowitch P., Nguéa P. A. and Enserink J. M. (2015). SUMO-regulated transcription: challenging the dogma. *BioEssays* 37, 1095-1105. 10.1002/bies.201500065 26354225

[BIO024679C7] Geiss-Friedlander R. and Melchior F. (2007). Concepts in sumoylation: a decade on. *Nat. Rev. Mol. Cell Biol.* 8, 947-956. 10.1038/nrm2293 18000527

[BIO024679C8] Hwang E. J., Lee J. M., Jeong J., Park J. H., Yang Y., Lim J.-S., Kim J. H., Baek S. H. and Kim K. I. (2009). SUMOylation of RORalpha potentiates transcriptional activation function. *Biochem. Biophys. Res. Commun.* 378, 513-517. 10.1016/j.bbrc.2008.11.072 19041634

[BIO024679C9] Kim J.-W., Yang H.-J., Oel A. P., Brooks M. J., Jia L., Plachetzki D. C., Li W., Allison W. T. and Swaroop A. (2016a). Recruitment of Rod photoreceptors from short-wavelength-sensitive cones during the evolution of nocturnal vision in mammals. *Dev. Cell* 37, 520-532. 10.1016/j.devcel.2016.05.023 27326930 PMC4918105

[BIO024679C10] Kim J.-W., Yang H.-J., Brooks M. J., Zelinger L., Karakülah G., Gotoh N., Boleda A., Gieser L., Giuste F., Whitaker D. T. et al. (2016b). NRL-regulated transcriptome dynamics of developing rod photoreceptors. *Cell Rep.* 17, 2460-2473. 10.1016/j.celrep.2016.10.074 27880916 PMC5131731

[BIO024679C11] Klenk C., Humrich J., Quitterer U. and Lohse M. J. (2006). SUMO-1 controls the protein stability and the biological function of phosducin. *J. Biol. Chem.* 281, 8357-8364. 10.1074/jbc.M513703200 16421094

[BIO024679C12] Lamb T. D. (2013). Evolution of phototransduction, vertebrate photoreceptors and retina. *Prog. Retin. Eye Res.* 36, 52-119. 10.1016/j.preteyeres.2013.06.001 23792002

[BIO024679C13] Liao Y., Smyth G. K. and Shi W. (2014). featureCounts: an efficient general purpose program for assigning sequence reads to genomic features. *Bioinformatics* 30, 923-930. 10.1093/bioinformatics/btt656 24227677

[BIO024679C14] Liu B., Mink S., Wong K. A., Stein N., Getman C., Dempsey P. W., Wu H. and Shuai K. (2004). PIAS1 selectively inhibits interferon-inducible genes and is important in innate immunity. *Nat. Immunol.* 5, 891-898. 10.1038/ni1104 15311277

[BIO024679C15] Liu Y.-Y., Kogai T., Schultz J. J., Mody K. and Brent G. A. (2012). Thyroid hormone receptor isoform-specific modification by small ubiquitin-like modifier (SUMO) modulates thyroid hormone-dependent gene regulation. *J. Biol. Chem.* 287, 36499-36508. 10.1074/jbc.M112.344317 22930759 PMC3476315

[BIO024679C16] Lyst M. J. and Stancheva I. (2007). A role for SUMO modification in transcriptional repression and activation. *Biochem. Soc. Trans.* 35, 1389-1392. 10.1042/BST0351389 18031228 PMC2871292

[BIO024679C17] Nacerddine K., Lehembre F., Bhaumik M., Artus J., Cohen-Tannoudji M., Babinet C., Pandolfi P. P. and Dejean A. (2005). The SUMO pathway is essential for nuclear integrity and chromosome segregation in mice. *Dev. Cell* 9, 769-779. 10.1016/j.devcel.2005.10.007 16326389

[BIO024679C18] Ng L., Hurley J. B., Dierks B., Srinivas M., Salto C., Vennström B., Reh T. A. and Forrest D. (2001). A thyroid hormone receptor that is required for the development of green cone photoreceptors. *Nat. Genet.* 27, 94-98. 10.1038/83829 11138006

[BIO024679C19] Ng L., Lu A., Swaroop A., Sharlin D. S., Swaroop A. and Forrest D. (2011). Two transcription factors can direct three photoreceptor outcomes from rod precursor cells in mouse retinal development. *J. Neurosci.* 31, 11118-11125. 10.1523/JNEUROSCI.1709-11.2011 21813673 PMC3158567

[BIO024679C20] Nikonov S. S., Kholodenko R., Lem J. and Pugh E. N. Jr (2006). Physiological features of the S- and M-cone photoreceptors of wild-type mice from single-cell recordings. *J. Gen. Physiol.* 127, 359-374. 10.1085/jgp.200609490 16567464 PMC2151510

[BIO024679C21] Oh E. C. T., Cheng H., Hao H., Jia L., Khan N. W. and Swaroop A. (2008). Rod differentiation factor NRL activates the expression of nuclear receptor NR2E3 to suppress the development of cone photoreceptors. *Brain Res.* 1236, 16-29. 10.1016/j.brainres.2008.01.028 18294621 PMC2660138

[BIO024679C22] Onishi A., Peng G.-H., Hsu C., Alexis U., Chen S. and Blackshaw S. (2009). Pias3-Dependent SUMOylation Directs Rod Photoreceptor Development. *Neuron* 61, 234-246. 10.1016/j.neuron.2008.12.006 19186166 PMC2701228

[BIO024679C23] Onishi A., Peng G.-H., Chen S. and Blackshaw S. (2010). Pias3-dependent SUMOylation controls mammalian cone photoreceptor differentiation. *Nat. Neurosci.* 13, 1059-1065. 10.1038/nn.2618 20729845 PMC2932661

[BIO024679C24] Roberts M. R., Hendrickson A., McGuire C. R. and Reh T. A. (2005). Retinoid X receptor (gamma) is necessary to establish the S-opsin gradient in cone photoreceptors of the developing mouse retina. *Invest. Ophthalmol. Vis. Sci.* 46, 2897-2904. 10.1167/iovs.05-0093 16043864

[BIO024679C25] Robinson M. D., McCarthy D. J. and Smyth G. K. (2010). edgeR: a Bioconductor package for differential expression analysis of digital gene expression data. *Bioinformatics* 26, 139-140. 10.1093/bioinformatics/btp616 19910308 PMC2796818

[BIO024679C26] Roger J. E., Nellissery J., Kim D. S. and Swaroop A. (2010). Sumoylation of bZIP transcription factor NRL modulates target gene expression during photoreceptor differentiation. *J. Biol. Chem.* 285, 25637-25644. 10.1074/jbc.M110.142810 20551322 PMC2919127

[BIO024679C27] Roger J. E., Hiriyanna A., Gotoh N., Hao H., Cheng D. F., Ratnapriya R., Kautzmann M.-A. I., Chang B. and Swaroop A. (2014). OTX2 loss causes rod differentiation defect in CRX-associated congenital blindness. *J. Clin. Invest.* 124, 631-643. 10.1172/JCI72722 24382353 PMC3904630

[BIO024679C28] Roth W., Sustmann C., Kieslinger M., Gilmozzi A., Irmer D., Kremmer E., Turck C. and Grosschedl R. (2004). PIASy-deficient mice display modest defects in IFN and Wnt signaling. *J. Immunol.* 173, 6189-6199. 10.4049/jimmunol.173.10.6189 15528356

[BIO024679C29] Rytinki M. M., Kaikkonen S., Pehkonen P., Jääskeläinen T. and Palvimo J. J. (2009). PIAS proteins: pleiotropic interactors associated with SUMO. *Cell. Mol. Life Sci.* 66, 3029-3041. 10.1007/s00018-009-0061-z 19526197 PMC11115825

[BIO024679C30] Santti H., Mikkonen L., Anand A., Hirvonen-Santti S., Toppari J., Panhuysen M., Vauti F., Perera M., Corte G., Wurst W. et al. (2005). Disruption of the murine PIASx gene results in reduced testis weight. *J. Mol. Endocrinol.* 34, 645-654. 10.1677/jme.1.01666 15956336

[BIO024679C31] Schmidt D. and Muller S. (2003). PIAS/SUMO: new partners in transcriptional regulation. *Cell. Mol. Life Sci.* 60, 2561-2574. 10.1007/s00018-003-3129-1 14685683 PMC11138616

[BIO024679C32] Seet B. T., Dikic I., Zhou M.-M. and Pawson T. (2006). Reading protein modifications with interaction domains. *Nat. Rev. Mol. Cell Biol.* 7, 473-483. 10.1038/nrm1960 16829979

[BIO024679C33] Sundvall M., Korhonen A., Vaparanta K., Anckar J., Halkilahti K., Salah Z., Aqeilan R. I., Palvimo J. J., Sistonen L. and Elenius K. (2012). Protein inhibitor of activated STAT3 (PIAS3) protein promotes SUMOylation and nuclear sequestration of the intracellular domain of ErbB4 protein. *J. Biol. Chem.* 287, 23216-23226. 10.1074/jbc.M111.335927 22584572 PMC3391121

[BIO024679C34] Swain P. K., Hicks D., Mears A. J., Apel I. J., Smith J. E., John S. K., Hendrickson A., Milam A. H. and Swaroop A. (2001). Multiple phosphorylated isoforms of NRL are expressed in rod photoreceptors. *J. Biol. Chem.* 276, 36824-36830. 10.1074/jbc.M105855200 11477108

[BIO024679C35] Swaroop A., Kim D. and Forrest D. (2010). Transcriptional regulation of photoreceptor development and homeostasis in the mammalian retina. *Nat. Rev. Neurosci.* 11, 563-576. 10.1038/nrn2880 20648062 PMC11346175

[BIO024679C36] Swindell E. C., Bailey T. J., Loosli F., Liu C., Amaya-Manzanares F., Mahon K. A., Wittbrodt J. and Jamrich M. (2006). Rx-Cre, a tool for inactivation of gene expression in the developing retina. *Genesis* 44, 361-363. 10.1002/dvg.20225 16850473

[BIO024679C37] Tahk S., Liu B., Chernishof V., Wong K. A., Wu H. and Shuai K. (2007). Control of specificity and magnitude of NF-kappa B and STAT1-mediated gene activation through PIASy and PIAS1 cooperation. *Proc. Natl. Acad. Sci. USA* 104, 11643-11648. 10.1073/pnas.0701877104 17606919 PMC1913887

[BIO024679C38] Trapnell C., Pachter L. and Salzberg S. L. (2009). TopHat: discovering splice junctions with RNA-Seq. *Bioinformatics* 25, 1105-1111. 10.1093/bioinformatics/btp120 19289445 PMC2672628

[BIO024679C39] Viets K., Eldred K. C. and Johnston R. J. Jr (2016). Mechanisms of photoreceptor patterning in vertebrates and invertebrates. *Trends Genet.* 32, 638-659. 10.1016/j.tig.2016.07.004 27615122 PMC5035628

[BIO024679C40] Widagdo J., Taylor K. M., Gunning P. W., Hardeman E. C. and Palmer S. J. (2012). SUMOylation of GTF2IRD1 regulates protein partner interactions and ubiquitin-mediated degradation. *PLoS ONE* 7, e49283 10.1371/journal.pone.0049283 23145142 PMC3493543

[BIO024679C41] Wong K. A., Kim R., Christofk H., Gao J., Lawson G. and Wu H. (2004). Protein inhibitor of activated STAT Y (PIASy) and a splice variant lacking exon 6 enhance sumoylation but are not essential for embryogenesis and adult life. *Mol. Cell. Biol.* 24, 5577-5586. 10.1128/MCB.24.12.5577-5586.2004 15169916 PMC419860

[BIO024679C42] Wu C.-S. and Zou L. (2016). The SUMO (Small Ubiquitin-like Modifier) ligase PIAS3 primes ATR for checkpoint activation. *J. Biol. Chem.* 291, 279-290. 10.1074/jbc.M115.691170 26565033 PMC4697162

[BIO024679C43] Yagil Z., Nechushtan H., Kay G., Yang C. M., Kemeny D. M. and Razin E. (2010). The enigma of the role of protein inhibitor of activated STAT3 (PIAS3) in the immune response. *Trends Immunol.* 31, 199-204. 10.1016/j.it.2010.01.005 20181527

